# Research on the factors influencing the re-purchase intention on short video platforms: A case of China

**DOI:** 10.1371/journal.pone.0265090

**Published:** 2022-03-15

**Authors:** Baodeng Lin, Yongyi Chen, Liping Zhang

**Affiliations:** 1 School of Economics and Trade, Fujian Jiangxia University, Fuzhou, China; 2 Institute of Education, Xiamen University, Xiamen, China; University of Oklahama Norman Campus: The University of Oklahoma, UNITED STATES

## Abstract

Short video platforms, which thrive along with the video-based consumption industry, have become a new channel adopted by an increasing number of enterprises to distribute products. Therefore, it is necessary to study the factors influencing the consumer’s intention to re-purchase on short video platforms, which is helpful for firms to maintain their competitiveness. This paper, based on the customer value theory, seeks to establish a structural model for such factors. The intermediary effect of customer loyalty on customer satisfaction and repurchase intention of customers is also analyzed. Questionnaires were distributed and collected from users of short video platforms in China. Results show that short video content, customer experience, and perceived value have positive impacts on customer satisfaction, and customer satisfaction, along with customer loyalty, exert positive impacts on repurchase intention. Notably, customer loyalty plays an intermediary role between customer satisfaction and repurchase intention. Based on the aforesaid findings, theoretical implications are discussed and managerial implications to increase repurchase rate are offered.

## Introduction

With the continuous development of 5G network, the mobile internet industry has undergone earth-shaking changes in the “era of short video” dominated by visual communication. As indicated in the 47th *Statistical Report on Internet Development in China* published by CNNIC, by December 2020, the number of online videos (including short video) users has reached 927 million, accounting for 93.7% of all netizens. Thereinto, the number of short video users has reached 873 million, accounting for 88.3% of netizens [1]. As the mobile internet develops rapidly, a large number of short video platforms have emerged, such as Tik Tok, Kwai, Pear Video, and MiaoPai. The video covers a wide range of contents from science popularization, to daily vlogs such as makeups, pets, cuisine preparation, etc. Among these platforms, “Tik Tok”, which is preferred by the young, and “Kwai”, which takes the route of “encircling cities from rural areas”, are the most popular.

In addition, these platforms enable the videos to be reposted, commented, shared, and liked, which caters to the demands for today’s mobile social networking. This phenomenon can be verified by the popularity of short video platforms among contemporary youth. Specifically, when people are in quarantine during the outbreak of COVID-19, there are an increasing number of users spending more time on various network video apps, which brings an unprecedented opportunity to short video shopping in China. However, it is critical to consider whether such a platform can retain customers long enough for re-purchase. Therefore, analyzing the influencing factors of repurchase willingness on the short video platform is of great importance.

In this paper, these factors are analyzed empirically based on previous studies to enrich the studies on the repurchase willingness in this industry. Finally, some suggestions are made based on the empirical analysis for related enterprises and platforms, so that they can better understand customers’ psychology in this regard so as to retain more loyal customers and achieve a higher re-purchase rate. In the meantime, new ideas are also proposed for the future development of short video platforms to strengthen their competitiveness.

## Literature review

### Short videos and related consumption activities

Short videos, which is typically a few seconds long, can be shot and edited on mobile terminals, and then shared promptly on social platforms. Zhang Haitao et al. (2020) defined it as fragmented entertainment featured with a low creation threshold and high sociability [2]. Along with the popularity of this mode of entertainment, video contents are increasingly diversified. Peng Lan (2019), indicated that such contents, containing people-oriented emotions and warm lifestyles, can carry forward the folk culture [3]. As stated in *2020 China Research Report on Internet Audio-Visual Development*, the number of short video users, which are dominated by the “post-80s” and “post-90s”, has reached 818 million, nearly accounting for 90% of all netizens. 60.4% of these users browse short videos daily. By June 2020, the users have involved more age brackets, but they are mostly the generation born in the 1980s and people with low or medium levels of education [4]. By comprehensively observing such users’ motivations, likes, dislikes and lifestyles, Zhang Tianli et al. (2019) found that many of them are pursuing for “energy, connection and individuation”, showing the characteristics of group image [5].

Due to the rapid expansion of social e-commerce, short video consumption has become a new force and channel of online shopping, and the products can be presented to the user in a more vivid and direct form. This has prompted many enterprises to promote their products via short videos on short video platforms. Wang Jianlei (2021) suggested that customers tend to have an enjoyable, innovative, fun, and immersive shopping experience when shopping on short video platforms. For these users, the purpose of short video consumption is to gain pleasure, value and feedback capabilities, which differs significantly from shopping on traditional media [6]. Zhou Xuanchen et al. (2021) pointed out that short video contents are characterized by fast pace and strong impact, which stimulates customers’ vision and sense. However, people will stop thinking about other things when they are immersed in the instantaneous sensory pleasure, which results in the fast-food style of short video consumption [7]. Cao Danning (2019) concluded that short video has changed from the simple media of one-way content output to the composite one of “content+function”, which strengthens the relationship between users and platforms and contributes to a new consumption pattern [8].

#### Connotation of repurchase intention

Feng Jianying et al. (2006) defined intention as the subjective probability of specific behaviors [9]. The stronger the intention a person has, the higher the probability he/she makes a corresponding behavior. Dodds et al. (1991) thought that purchase intention is not only the subjective probability or possibility when a customer buys a particular product, but also his/her subjective consciousness and potency on a psychological level [10].

Re-purchase is also known as “repeated purchase”. Chen Mingliang (2002) proposed in his research that, re-purchase intention means that customers wish and tend to maintain a trading relationship with suppliers [11]. Harrison (2001) considered it as the intensity of actual repurchasing behavior. The customers with a stronger re-purchase intention will be more likely to buy the same thing [12]. The relationship between such behavior and intention is in essence the relation between behavior and consciousness. In a sense, therefore, the re-purchasing behavior depends on the re-purchase intention, and it can be promoted by studying the influencing factors of this intention. Thus, the short video consumption industry can develop rapidly.

#### Research on effects of short videos on purchase intention

The short video, with a huge potential market value, can stimulate customers’ purchase intention. Wei Jingqiu et al. (2020) applied the SOR Framework to their analysis and found that users like to browse the short video mainly because of its serviceability, entertainment, and usability. These factors will directly affect customers’ emotional experience and thus stimulate their purchase intention and behavior [13]. Wang Xiangning (2020) concluded that users will be more willing to buy a brand after they develop a closer relationship with this brand under the effect of short video contents [14]. Besides, such contents are more influential than those from the traditional advertisement in aspects of entertainment, function, and social interaction, and these factors also affect customers’ purchase intention.

### Customer value theory

Customers will measure the advantages and disadvantages of a product or service before buying it, which is known as customer values. Woodruff (1997) explained this concept from the perspective of changing customers’ values. In his opinion, customer values refer to customers’ satisfaction with a product’s attributes and auxiliary functions based on their likes and dislikes. Li Kouqing (2001) defined it as the benefit received by customers from an enterprise that participates in their consuming behavior by managing them [15]. Therefore, the customer value theory, which extends customer-guided marketing, is the footstone to improve customer satisfaction and establish customer loyalty. Philip Kotler (1972) believed that for enterprises, customer value management mainly lies in how to build a long-term interest relationship with customers, and perceived values of customers are the key to greater competitive advantages [16]. To this end, enterprises shall improve their services and resources and provide customers with high-quality products or services, thus maintaining such value.

### Preliminary summary

In this chapter, a literature review about short video consumption and repurchase intention is presented. Although previous studies laid the theoretical basis for this paper, gaps still exist. First, those studies primarily focus on short video consumption, and only a few of them involve the influencing factors of re-purchase. Second, there are some follow-up problems faced by the short video consumption industry, that is, how to maintain a good relationship with customers after their purchases and how to improve the loyalty of existing customers. Therefore, this paper analyzes the repurchase intention and factors influencing it and studied the interrelationship among these factors. In addition, an empirical analysis is conducted to study how such intention relates to each factor in the context of the internet, so that the enterprises in this industry can work out better marketing strategies to promote customers’ repurchasing behavior.

## Research hypothesis

### Effect of short video contents on the repurchase intention towards short video consumption

Customers’ perception or impression of a product is directly influenced by the interestingness or authenticity of short video contents. In the era of the internet, the qualities of contents vary, so short video marketers need to know how to stand out from others. Leal et al. (2014) concluded that User-generated content affects consumer decision-making through social influence factors [17]. As short videos are limited in length, the selling point of products becomes especially prominent during the shooting process. In addition, such video can be shot at a low cost and spread at a high speed to achieve better promotional effects. Compared with traditional advertisements that comply with strict regulations on contents, short videos have weird, funny, instructional, and even vulgar contents to attract customers and stimulate their purchase intention. Furthermore, customers will have a fixed impression of a product or brand and get psychologically satisfied again during re-purchase. Thus, the group image can be formed in this product or brand to affect customers. Based on what was stated above, the following hypothesis is put forward for the relationship between short video contents and customer satisfaction:

H1: Short video contents have a positive impact on customer satisfaction.

### Effect of customer experience on the repurchase intention towards short video consumption

Customer experience refers to “customers’ activities to create value for a product or service through their firsthand experience, thus enhancing customer and exchange values under particular circumstances”. We defined it as customers’ perception and emotion arising from their interaction with a product or service under specific consumption conditions. Li Qigeng et al. (2011) concluded that the repurchase intention will be positively affected by the brand experience in sense, emotion, cognition and relation [18]. Zhou Shouliang et al. (2019) also thought that the information quality and perceived interactivity of short videos can determine customers’ confidence in a product or service. Therefore, it is critical to strengthen their trust and loyalty [19]. Customer experience emphasizes customers’ subjective sensation from browsing products to asking for after-sales services, and the satisfaction is directly influenced by their experience before and after the purchase. Besides, while shopping through short videos, customers will integrate their own perceptions and feelings with video effects. Hence, the short video experience is closely linked to the repurchase intention. In accordance with the above research, it can be hypothesized that:

H2: Customer experience has a positive impact on customer satisfaction.

### Effect of perceived values of customers on the repurchase intention towards short video consumption

*Perceived values of* customers, are defined by Zaithaml as the “overall assessment made by customers on the usefulness of a product or service after weighing perceived benefits and consumption costs”. Lin et al. (2005) indicated that the reuse intention is positively affected by perceived enjoyment and usefulness, and the expected degree of confirmation [20]. Wang Dunhai (2018) pointed out that this perceived enjoyment will promote the repurchase intention, and there is a mutual regulation relation between *perceived values of* customers and repurchase intention [21]. Therefore, it can be seen that customers will have a higher satisfaction if an enterprise or brand can create a higher perceived value for them by presenting its products in all respects through short videos. Thus, customers will be more willing to trust this enterprise or brand and repurchase its products. Due to the relevant sales connection between customers and their perceived values, the sense of belonging will be enhanced if they can perceive values of products in advance. As indicated by foreign and domestic scholars, the perceived value is positively correlated with customer satisfaction and customer loyalty. When such values are higher than expected, the satisfaction and repurchase intention will be enhanced. Conversely, the satisfaction and intention will decline. These lead to the following hypothesis:

H3: Perceived values of customer have a positive impact on customer satisfaction.

### Effect of customer satisfaction on the repurchase intention on short video platforms

Philip Kotler (1995) defined customer satisfaction as “a person’s feeling of pleasure or disappointment when he/she compares the perceived outcome of a product or service against his/her expectations” [22]. The more the customers are satisfied with the products they have bought, the higher the satisfaction will be and the more they will trust in and rely on the enterprise or brand. Xing Wenxiang (2014) thought that customers’ repurchasing behavior is influenced by various factors, such as product diversification, individuation, quality, post-purchase evaluation, brand reputation, payment, distribution, etc., of which customer satisfaction is the most crucial one [23]. Furthermore, customer satisfaction is also considered to be an important factor affecting customer loyalty. As indicated by Wang Chunxiao et al. (2003), this factor, which is an essential prerequisite for loyalty, can significantly affect customers’ behavioral loyalty [24]. Li Siman (2009) regarded it as a vital factor affecting the repurchase intention because customers’ purchasing behavior depends on customer satisfaction [25]. In view of the above, the following hypotheses are proposed:

H4a: Customer satisfaction has a positive impact on customer loyalty.
H4b: Customer satisfaction has a positive impact on the repurchase intention on short video platforms.

### Effect of customer loyalty on the repurchase intention on short video platforms

Customer loyalty refers to the mental tendency that a customer trusts an enterprise or brand after buying its products and wants to repurchase such products on an ongoing basis. Oliver (1997) optimized this definition and theoretically proposed four stages for forming customer loyalty, namely cognitive, attitudinal, emotional and behavioral loyalties [26]. Chen Mingliang indicated that customer loyalty is determined by perceived values, transfer cost, customer confidence and customer satisfaction [27]. In all circumstances, customer loyalty can be reflected in repurchasing behavior. The higher customer loyalty is, the stronger the repurchase intention will be. Therefore, loyalty is another psychological index for such intention. Lv Xiaoping (2008) pointed out that e-loyalty is affected by e-satisfaction and e-trust. In other words, a customer will not reuse a network platform unless he/she trusts and be satisfied with it [28]. Generally, customer loyalty, which is also an influencing factor of short video consumption, will increase with customer satisfaction. Only when a customer is satisfied with a product or service to a certain extent will he/she be faithful to it. Therefore, the following hypotheses are made:

H5: Customer loyalty has a positive impact on the repurchase intention towards short video consumption.
H6: Customer loyalty plays an intermediary role between customer satisfaction and repurchase intention.

### Research model

**Fig 1:**
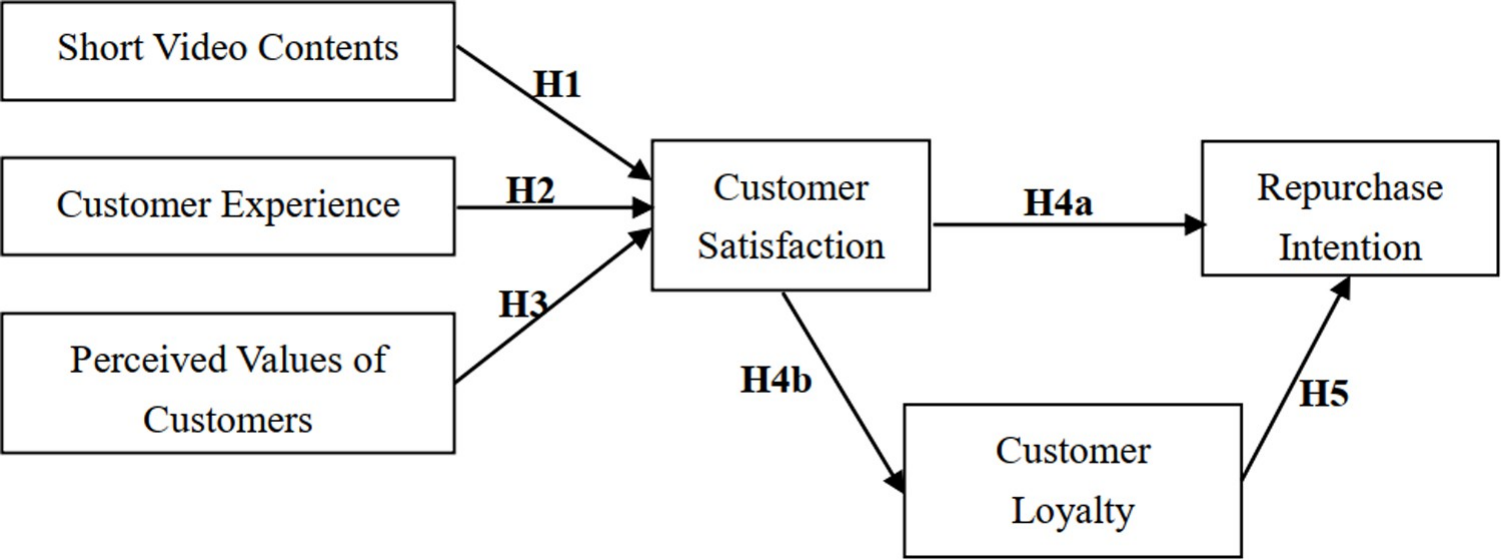
Influencing factors of the repurchase intention on short video platforms.

Based on the literature review above and the actual situation, a model for the influencing factors of repurchase intention on short video platforms is constructed based on the customer value theory. As shown in Fig 1, this model contains five explanatory/independent variables (customer satisfaction, perceived value, experience, loyalty and short video contents) and one outcome/dependent variable (repurchase intention).

### Questionnaire design

In this research, the questionnaire includes three parts: part one focuses on the basic information of respondents and their attitude towards shopping on short video platforms; and part two, which is the core of this questionnaire, focuses on fifteen measurement items about the above five explanatory variables. Thereinto, the 5-Point Likert Scale was adopted for the survey; and the items about the influencing factors of repurchase intention towards short video consumption referred to foreign and domestic questionnaires conducted previously in this respect, but some adjustments were made in accordance with the research model in this paper. Table 1 presents the questionnaire in detail.

**Table 1:** Variables and measurement items.

Variable	No.	Measurement item	References
Short video content	IMV1	I will buy a product if its short video can show how good it is.	Zhou Xingyu (2019) [29]
	IMV2	I will buy a product if its short video can stimulate my shopping desire.	
	IMV3	Compared with the graphic introduction on other shopping platforms, the short video enables me to experience the product function in a better way.	
Customer experience	UE1	I think it is highly efficient to shop on short video platforms.	Ducoffe R H (1996) [30]
	UE2	The short video apps will recommend the goods that I may interest in according to my browsing history.	
	UE3	Compared with other like products, those sold on short video platforms have higher cost performance.	
Perceived values of Customers	CPV1	I think the products sold on short video platforms have good performance.	Zeithaml VA (1996) and Li Jiamin (2019) [31, 32]
	CPV2	I think the products sold on short video platforms have high quality.	
	CPV3	I think the products sold on short video platforms are reliable.	
Customer satisfaction	CS1	I’m satisfied with the shopping on short video apps.	Ni Hongyao (2013) [33]
	CS2	I think it wise to buy products on short video apps.	
	CS3	I’m more satisfied with the shopping on short video apps than on other platforms.	
Customer loyalty	CL1	Compared with other shopping platforms, this short video app is used by me most frequently.	Chen Mingliang (2003) and the author [27]
	CL2	I prefer short video apps to other shopping platforms.	
	CL3	I will keep using this short video app if I have no special need.	
Repurchase intention	RI1	I’d like to use short video platforms for a long time.	Phillip et al. (2003) [34]
	RI2	I’d like to recommend my friends to shop on short video platforms.	
	RI3	If necessary, it is likely for me to buy other products on short video platforms.	

### Data collection and analysis

#### Data collection of questionnaire survey

This questionnaire survey, lasting from March to May 2021, was conducted by a random sampling method. The questionnaire was posted on the website of wjx.cn through WeChat for respondents who shopped on short video platforms at least once in China. Finally, a total of 208 questionnaires were received, with 199 effective ones, and the response rate was 95.7%. SEM, as other statistical techniques, requires an appropriate sample size in order to produce reliable estimates [35]. Harris and Schaubroeck proposed that a sample size of about 200 could guarantee robust structural equation modeling [36].

The sample data obtained were analyzed with SPSS19.0 and AMOS 17.0.

### Descriptive statistical analysis

From Table 2, it can be seen that the respondents were all highly educated, and 75.38% of them had bachelor degrees or above. Besides, the female accounted for 51.25%; and the respondents aged 18–25 accounted for 66.33%. Most respondents were students or enterprise employees. Thereinto, 63.82% of them spent less than 100 yuan on short video consumption monthly, while 27.14% of them spent 100–500 yuan. Statistical results also show that the most common short video apps include Tik Tok, Kwai, and Bilibili.

**Table 2:** Demographic characteristics of samples.

Basic information	Item	Frequency	Proportion
Gender	Male	97	48.74%
	Female	102	51.25%
Age (yr)	Under 18	5	2.51%
	18–25	132	66.33%
	26–40	48	24.12%
	41 or above	14	7.03%
Educational background	Senior high school, technical secondary school or below	23	11.56%
	Junior college	26	13.07%
	Bachelor degree	139	69.85%
	Master degree or above	11	5.53%
Occupation	Students	99	49.75%
	Employees of enterprise units or public institutions	63	31.66%
	Officers of government agencies	12	6.03%
	Freelancers	14	7.04%
	Others	11	5.53%
Monthly amount spent on short video platforms	Less than 100 yuan	127	63.82%
	100–500 yuan	54	27.14%
	500–1000 yuan	14	7.04%
	More than 1000 yuan	4	2.01%

### Measurement model test

First, the reliability of sample data was tested. According to the results shown in Table 3, the coefficients of Cronbach’s Alpha all exceed 0.7 in the total scale and each subscale, which means that the data of this questionnaire are reliable. Second, the discriminant validity of sample data was tested, as shown in Table 4. It can be seen that in the six columns, the AVE square roots are 0.715, 0.676, 0.859, 0.735, 0.839, and 0.752, respectively, which are higher than the values of other variables. This indicates that these sample data have good discriminant validity. Last, the convergent validity of sample data was tested. As presented in Table 5, the factor loading values are higher than 0.7 in respect of customer satisfaction, perceived value, experience, loyalty, and short video content, which shows that the questionnaire items are highly representative for variables. Besides, for each variable, the value of AVE is higher than 0.5, and that of composite reliability (CR) is higher than 0.7. Therefore, the model has good internal consistency and convergent validity.

**Table 3:** Results of reliability testing.

Variable	Number of items	Cronbach Alpha
Short video contents	3	0.754
Customer experience	3	0.714
Perceived values of customers	3	0.891
Customer satisfaction	3	0.780
Customer loyalty	3	0.875
Repurchase intention	3	0.789
Total scale	18	0.904

**Table 4:** Results of discriminant validity testing.

	Short video content	Customer experience	Perceived values of customers	Customer satisfaction	Customer loyalty	Repurchase intention
Short video content	0.715					
Customer experience	0.374	0.676				
Perceived values of customers	0.405	0.413	0.859			
Customer satisfaction	0.412	0.382	0.552	0.735		
Customer loyalty	0.308	0.358	0.551	0.495	0.839	
Repurchase intention	0.329	0.442	0.469	0.493	0.507	0.752

**Table 5:** Results of convergent factor loading.

Path			Estimate	AVE	CR
IMV3	IMV3	Short video contents	0.675	0.604	0.820
IMV2	<—	Short video contents	0.833		
IMV1	<—	Short video contents	0.814		
CS1	<—	Customer satisfaction	0.673	0.537	0.774
CS2	<—	Customer satisfaction	0.848		
CS3	<—	Customer satisfaction	0.662		
UE3	<—	Customer experience	0.821	0.654	0.850
UE2	<—	Customer experience	0.812		
UE1	<—	Customer experience	0.793		
CPV3	<—	Perceived values of customers	0.648	0.533	0.772
CPV2	<—	Perceived values of customers	0.720		
CPV1	<—	Perceived values of customers	0.812		
CL3	<—	Customer loyalty	0.784	0.672	0.860
CL2	<—	Customer loyalty	0.860		
CL1	<—	Customer loyalty	0.814		
RI1	<—	Repurchase intention	0.771	0.578	0.805
RI2	<—	Repurchase intention	0.735		
RI3	<—	Repurchase intention	0.775		

### Structural model analysis

#### Model fitting analysis

The structural model fit the data well based on fit statistics as can be seen in Table 6. The value of X2/df is 1.576, which is less than 3, showing an ideal matching degree; and the value of RMSEA is 0.054, which is less than 0.08, also showing an ideal matching degree. Meanwhile, other coefficients are higher than 0.9, which indicates a good degree of fitting. Therefore, this model can fit the data well on the whole.

**Table 6:** Overall fitting coefficients.

X2/df	RMSEA	GFI	AGFI	CFI	IFI	TLI
1.576	0.054	0.901	0.892	0.957	0.957	0.947

#### Structural model test

**Fig 2:**
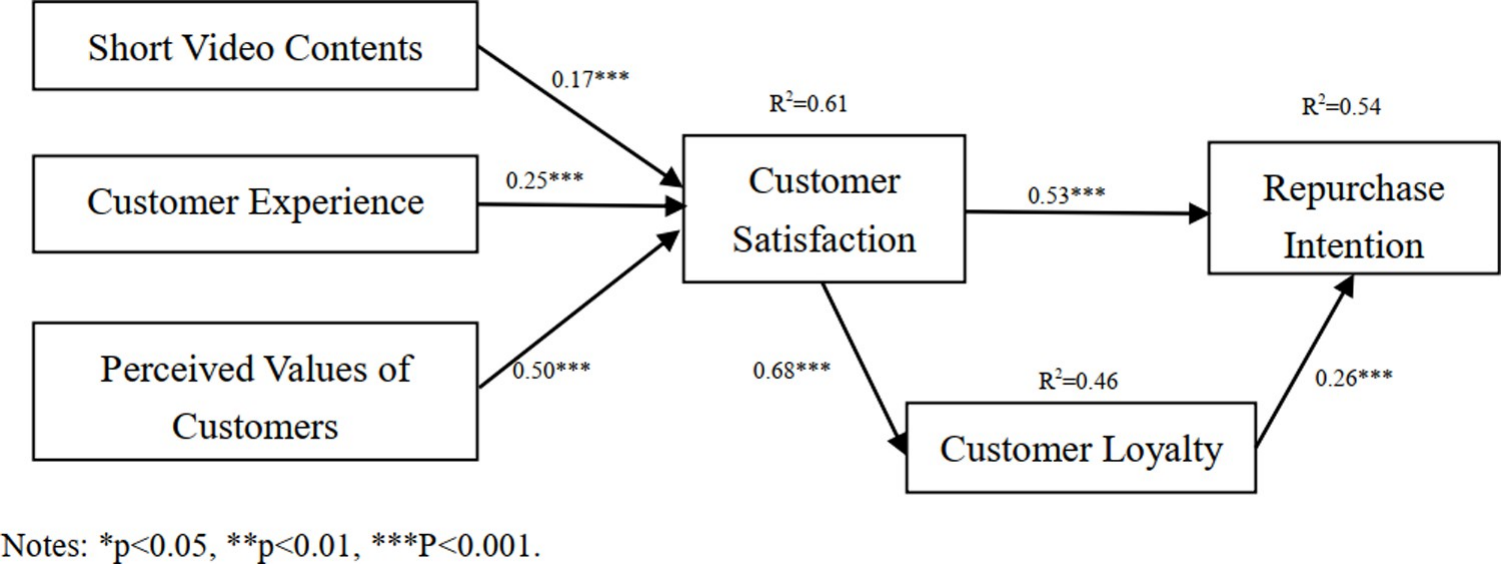
Results of path analysis in the structural model test. Notes: *p<0<0.05, **p<0.01, ***p<0.001.

The structural model was tested to get the regression coefficients of standardized paths. As shown in Fig 2, the main effects are significantly verified in this model. Thereinto, short video content, customer experience, and perceived values have positive impacts on the satisfaction; and customer satisfaction and customer loyalty have positive influences on repurchase intention. These results are consistent with the research hypotheses of this paper.

As can be seen form the above data obtained from the analysis, the results of parameter estimation are ideal. The loading coefficients of latent variables and measurement indexes are higher than 0.5, which indicates that the path coefficients of such variables are significant. Table 7 presents the empirical results. It can be known from the path analysis that the satisfaction is positively influenced by short video content (β = 0.17, p<0.001), customer experience (β = 0.25, p<0.001) and perceived values of customers (β = 0.50, p<0.001), which are consistent with the hypotheses of H1, H2 and H3. Meanwhile, the repurchase intention is positively influenced by customer satisfaction (β = 0.53, p<0.001) and customer loyalty (β = 0.26, p<0.001), which are consistent with the hypotheses of H4a and H5.

**Table 7:** Results of hypothesis testing.

Hypothesis	Interrelationship of variables	Standardized path	Test result
H1	Short video contents → customer satisfaction	0.17	Accepted
H2	Customer experience → customer satisfaction	0.25	Accepted
H3	Perceived values of customers → customer satisfaction	0.50	Accepted
H4a	Customer satisfaction → repurchase intention	0.53	Accepted
H4b	Customer satisfaction → customer loyalty	0.68	Accepted
H5	Customer loyalty → repurchase intention	0.26	Accepted

#### Analysis of intermediary effect

A multilevel regression analysis was conducted on customer satisfaction (independent variable), loyalty (intervening variable) and repurchase intention (dependent variable) to verify the loyalty’s intermediary effect between the satisfaction and such intention. The results are shown in Table 8.

**Table 8:** Results of the regression analysis on customer loyalty, satisfaction and repurchase intention.

	Loyalty		Repurchase intention		Repurchase intention	
	B	Std Error	B	Std Error	B	Std Error
Satisfaction	0.578***	0.089	0.545***	0.068	0.437***	0.073
Loyalty					0.187***	0.053
R-square	0.176		0.246		0.292	
Adjusted R-square	0.172		0.243		0.284	
F	42.167***		64.402***		40.364***	

Notes

**p<0.01

***P<0.001.

According to Table 8, the regression coefficient of customer satisfaction (independent variable) to repurchase intention (dependent variable) is significant; the hierarchical regression coefficients of customer satisfaction and customer loyalty (intervening variable) to repurchase intention are significant, and the regression coefficient of the satisfaction to the loyalty is also significant. Thereinto, the value of P is less than 0.001. Thus, it can be deduced that the loyalty plays an intermediary role between the satisfaction and such intention. That is, the repurchase intention can be affected by both satisfaction and loyalty. Therefore, the hypothesis (H6) stands, that is, customer loyalty plays an intermediary role between satisfaction and repurchase intention.

## Conclusions and management inspiration

### Research conclusions

In this paper, a structural model for the repurchase intention towards short video consumption is built and empirically tested. The model has five antecedent variables, including short video contents, customer experience, perceived value, satisfaction, and loyalty. One outcome variable, i.e., the repurchase intention, was included. Then, the following conclusions are drawn:

Short video content, customer experience, and perceived values of customers can regulate customer satisfaction to different degrees, showing significant positive impacts. In this paper, a structural equation model is built to analyze the interrelationship of variables. Results show that the customers’ satisfaction with short video consumption is positively affected by short video content, customer experience and perceived value, which show the standardized path coefficients of 0.17, 0.25, and 0.50, respectively. Among them, the perceived values of customers are the most influential variable, which are successively followed by customer experience and short video content. To some degree, customers can shop in a more dynamic and convenient way through short video consumption. Therefore, an enterprise shall provide valuable product information while interacting with its customers by means of short videos. Besides, this enterprise can help customers solve any problem of quality and usage in the same way, thereby improving customer satisfaction and strengthening the repurchase intention.

Customer satisfaction and loyalty have a significant positive impact on the repurchase intention on short video platforms. Thereinto, customer satisfaction is the most important determinant. In other words, a customer, who is satisfied with the products bought on short video platforms, will be more willing to repurchase them. Conversely, he/she may not buy them again on these platforms but resort to other shopping platforms. In addition, customer satisfaction can also positively affect customer loyalty. That is, customer loyalty will enhance when customer satisfaction reaches a certain degree. Thus, the customers, with higher loyalty, would tend to repurchase on the same platform.

The intermediary effect test shows that customer loyalty plays an intermediary role between customer satisfaction and repurchase intention. Thereinto, customer satisfaction means that customers’ expectations can be met by a product or service; and customer loyalty means that they want to buy this product or service again. Therefore, customer loyalty can be enhanced by improving customer satisfaction, thus strengthening the repurchase intention of customers on short video platforms.

### Management inspirations

#### To use the opinion leaders: “Label” the products to increase their appeal

Due to group psychology, the opinion leaders of short videos will influence customers’ consumption decisions and attitudes, thus igniting their enthusiasm for shopping. When a customer identifies with the brand image established by opinion leaders, such as stars or online celebrities, the consumption guide with high identifiability and reliability will be formed in his/her mind. In view of this, the merchants can take full advantage of such leaders’ strong influence and appeal and select them as short video publishers. When a product is “labeled” as an objective and positive one by these leaders, customers will be more confident in and faithful to this product and more willing to repurchase it.

#### To improve the visual presentation: Achieve customers’ immersive experience to develop their “empathy”

Customers will be immersed in short videos due to their visual presentation of different content. So, the content shall originate from and integrate with the elements of our daily life, such as creating some related scenes, to endow the products with cordial feeling and visual attractiveness. In this way, the content will be more real and closer to life, showing a creative point to attract customers. In addition, as customers tend to empathize with on-site and reportorial contents, the video shall present personal colors and true feelings to impress them and strengthen their purchase intention. At last, the contents shall be designed in accordance with customers’ psychological activities so as to improve their identification with products and to stimulate their shopping desire.

#### To meet customers’ needs: Enhance the perceived value and improve the sense of identity

Short videos should faithfully introduce products’ quality, performance, advantages and differences because customers tend to have a higher level of perceived values of the products with excellent qualities and reasonable prices. At the same time, all unnecessary expenses should be avoided so that customers’ actual perception is close to or even higher than their pre-purchase expectation. Besides, enterprises can provide new services to meet customers’ personalized demands, thus maintaining regular customers and attracting new ones. Only when customers’ needs are met, will the satisfaction be improved. Finally, enterprises shall post videos constantly and design content according to customers’ psychological activities. Thus, customers, with a stronger sense of identity, will satisfy with the products. Furthermore, enterprises shall establish a stable relationship with customers to strengthen their interactive perception of the video. When customers become more dependent on short video consumption, they will have a stronger desire to repurchase.

#### To optimize the platform mechanism: Boost customers’ confidence in products and reassure them

Short video platforms need to be upgraded continuously based on customers’ needs. In addition, improvements in commodity browsing experience, advertising promotion, and after-sales service are needed to enhance user stickiness. Besides, the management should be reinforced to improve products’ cost performance and crack down on counterfeits so that customers have more faith in such platforms. In this way, perceived values of customers can be maximized. For platforms, other improvements can be made in aspects such as page design, functions including search and payment, simplifying the shopping process, as well as shopping experiences including the efficiency, safety, and stability of online transactions. In this way, customers will recognize and trust the platforms and have stronger repurchase intention on these short video platforms.

#### Limitations and direction for future research

This study has certain limitations. The influence process of short video consumption on repurchase intention is very complicated. This study only focuses on customer satisfaction and customer loyalty, and fails to conduct a comprehensive and systematic analysis of the motivation and other antecedent variables of repeat purchases. For example, there are many factors that affect the repeated purchase of short video consumption, such as conversion costs, customer trust, and so on. Future research can explore the reconstruction intention of short video consumption from more perspectives. Similarly, limited by the setting of the research model, it is not possible to disassemble and verify the mechanism of internal factors such as consumers’ gender, income, education level, and external factors such as the category of short video platforms in more detail. The research model can be supplemented in future studies.

## Supporting information

S1 Appendix (DOCX)

S1 Data (SAV)
