# Supplementary material for: Research on the factors influencing the re-purchase intention on short video platforms: A case of China
Source: PLoS One. 2022 Mar 15;17(3):e0265090. doi: 10.1371/journal.pone.0265090 (PMC8923463; doi:10.1371/journal.pone.0265090)
Supplement: S1 Appendix — (DOCX) [file pone.0265090.s001.docx]

Questionnaire on Influencing Factors of Short Video Consumption Repurchase Willingness

Dear Sir and Madam:

Hello! Thank you for taking time out of your busy schedule to take part in this survey! This activity is mainly used to investigate the influencing factors of short video consumption repurchase intention. This survey is conducted anonymously, the results of the survey are for academic research purposes only, and the personal information involved will be kept strictly confidential. There is no right or wrong answer, please choose the most suitable answer according to the actual situation and real feelings. thank you very much!

**The first part basic information**

your gender? [Multiple choice] *

○Male

○Female

2. What is your age group? [Multiple choice] *

○Under 18 years old

○18-25 years old

○26-40 years old

○41 years old and above

3. What is your education level? [Multiple choice] *

○High school, secondary school or below

○ College

○Undergraduate

○Graduate and above

4. What is your occupation? [Multiple choice] *

○School students

○Employees of business units or institutions

○Government agency staff

○ Freelancers

○Other

**The second part is the perception of short video consumption**

1. Which short video APPs have you come into contact with or used? [Multiple Choice Questions] *

□Tik Tok

□quick hand

□bilibili

□Volcano video

□Toutiao video

□watermelon video

□Other

2. Have you ever shopped or consumed on short video apps? [Multiple choice] *

○Yes

○No

3. How much do you spend on the short video app every month? [Multiple choice] *

○ RMB 100 and below

○100-500 yuan

○500-1000 yuan

○More than 1000 yuan

4. I have some knowledge about short video APP shopping [single choice] *

Very unfamiliar ○1 ○2 ○3 ○4 ○5 Very well

Part III Influencing Factors of Short Video Consumption Repurchase Willingness

1. Compared with the graphic introduction of other shopping platforms, the video of the short video APP will make people experience the function of the product more [single choice] *

Strongly disagree ○1 ○2 ○3 ○4 ○5 Strongly agree

2. The short video of the product fully demonstrates how useful this product is, I would like to buy it [single choice] *

Strongly disagree ○1 ○2 ○3 ○4 ○5 Strongly agree

3. The video content on this short video can stimulate my shopping desire [single choice] *

Strongly disagree ○1 ○2 ○3 ○4 ○5 Strongly agree

4. Compared with similar products, I think the same product with short videos is more cost-effective [Multiple Choice] *

Strongly disagree ○1 ○2 ○3 ○4 ○5 Strongly agree

5. The short video app will recommend products that are suitable for me or that I am interested in based on my browsing traces [single choice] *

Strongly disagree ○1 ○2 ○3 ○4 ○5 Strongly agree

6. I think buying on short videos is an efficient way to buy [single choice] *

Strongly disagree ○1 ○2 ○3 ○4 ○5 Strongly agree

7. I think the product performance of the short video platform is very good [single choice] *

Strongly disagree ○1 ○2 ○3 ○4 ○5 Strongly agree

8. I think the products on the short video are of high quality [single choice] *

Strongly disagree ○1 ○2 ○3 ○4 ○5 Strongly agree

9. I think the products on the short video app are trustworthy [single choice] *

Strongly disagree ○1 ○2 ○3 ○4 ○5 Strongly agree

10. I am satisfied with the shopping experience on the short video APP [single choice] *

Strongly disagree ○1 ○2 ○3 ○4 ○5 Strongly agree

11. I think it’s wise for me to shop on short videos [single choice] *

Strongly disagree ○1 ○2 ○3 ○4 ○5 Strongly agree

12. Compared with other shopping platforms, I am more satisfied with shopping with short video apps [Multiple Choice] *

Strongly disagree ○1 ○2 ○3 ○4 ○5 Strongly agree

13. Compared with other shopping platforms, I have made the most purchases on this short video APP [single choice] *

Strongly disagree ○1 ○2 ○3 ○4 ○5 Strongly agree

14. Compared with other shopping platforms, I would prefer short videos for consumption [single choice] *

Strongly disagree ○1 ○2 ○3 ○4 ○5 Strongly agree

15. When there is no special need, I will always use this short video APP [single choice] *

Strongly disagree ○1 ○2 ○3 ○4 ○5 Strongly agree

16. I am very willing to continue to use the short video APP for a long time [single choice] *

Strongly disagree ○1 ○2 ○3 ○4 ○5 Strongly agree

17. I would like to recommend to my friends to use the short video platform for shopping [single choice] *

Strongly disagree ○1 ○2 ○3 ○4 ○5 Strongly agree

18. If necessary, I will most likely buy other products on the short video again [Multiple Choice]*

Strongly disagree ○1 ○2 ○3 ○4 ○5 Strongly agree
